# Supplementary figures and images for: Centriolar cap proteins CP110 and CPAP control slow elongation of microtubule plus ends
Source: J Cell Biol. 2025 Jan 23;224(3):e202406061. doi: 10.1083/jcb.202406061 (PMC11756378; doi:10.1083/jcb.202406061)

SourceDataF4

SourceDataF4C

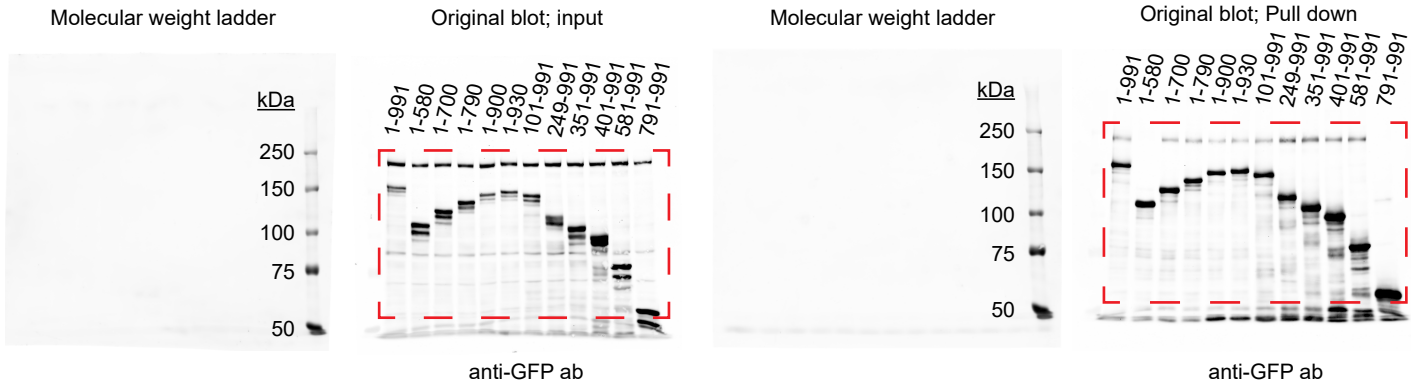

SourceDataF4D

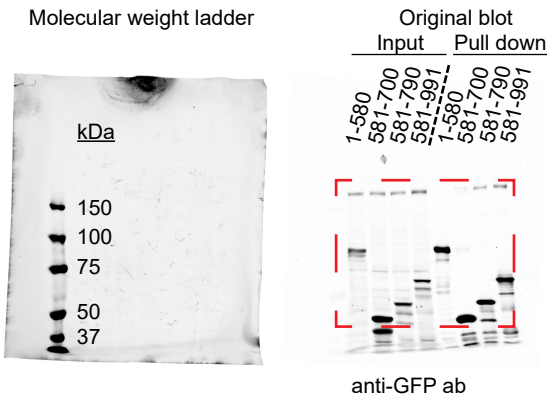

SourceDataF4E

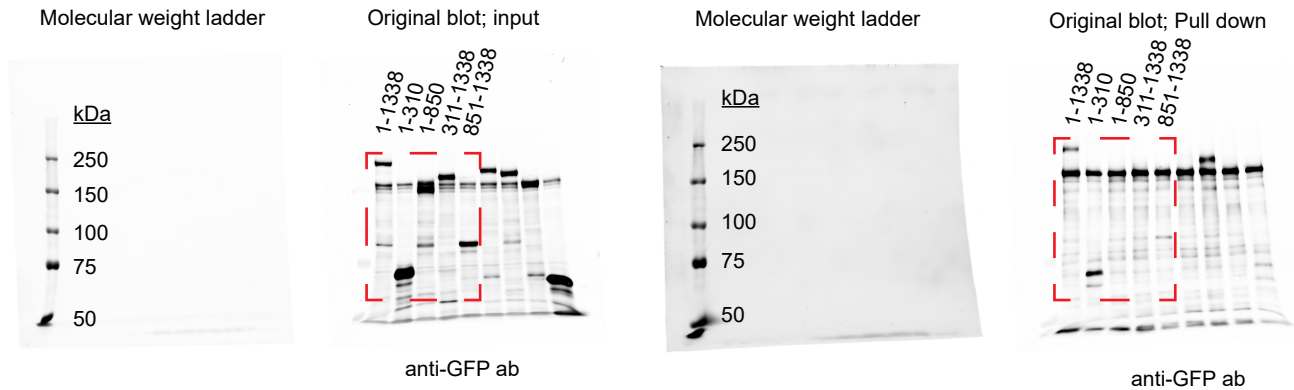

SourceDataF4F

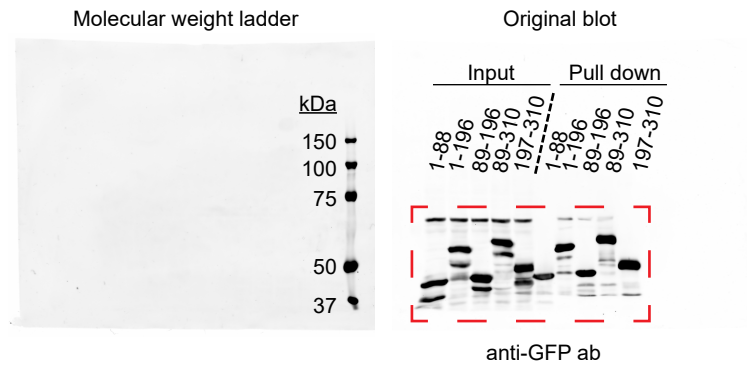

Supplement: SourceData F4 — is the source file for Fig. 4. [file jcb_202406061_sourcedataf4.pdf]

SourceDataF5

SourceDataF5G

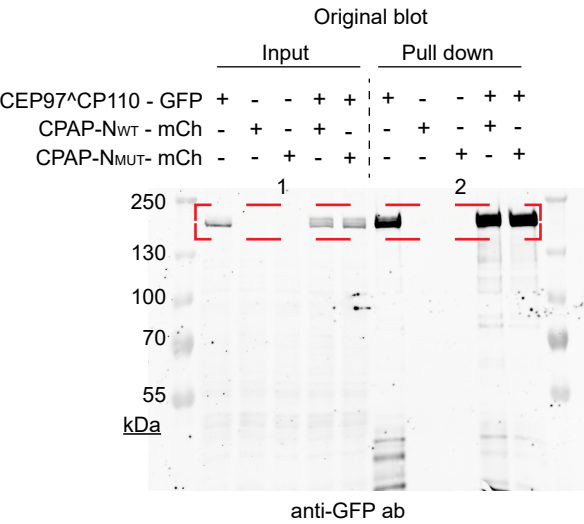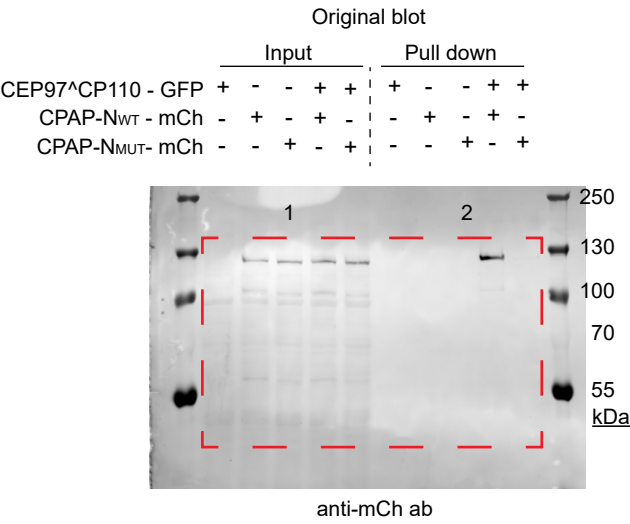

Supplement: SourceData F5 — is the source file for Fig. 5. [file jcb_202406061_sourcedataf5.pdf]

**SourceDataFS1A**

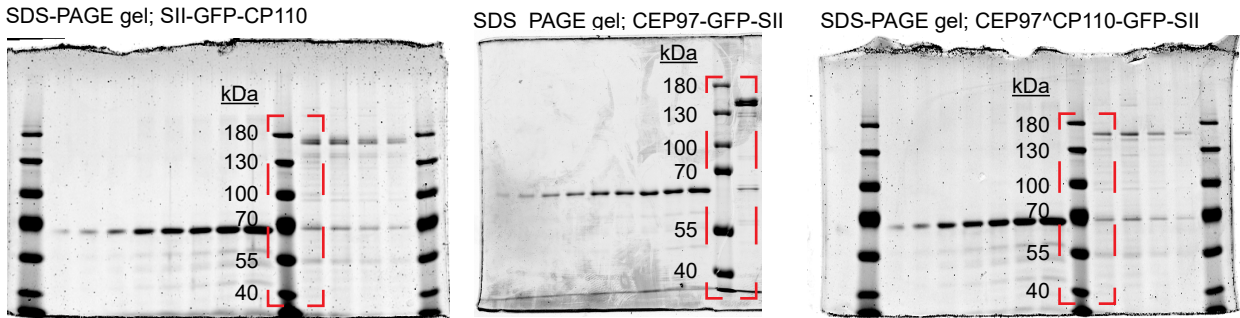

**SourceDataFS1F**

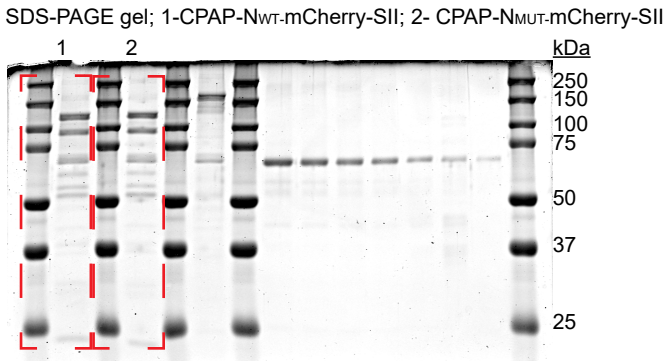

Supplement: SourceData FS1 — is the source file for Fig. S1. [file jcb_202406061_sourcedatafs1.pdf]

SourceDataFS5

SourceDataFS5A

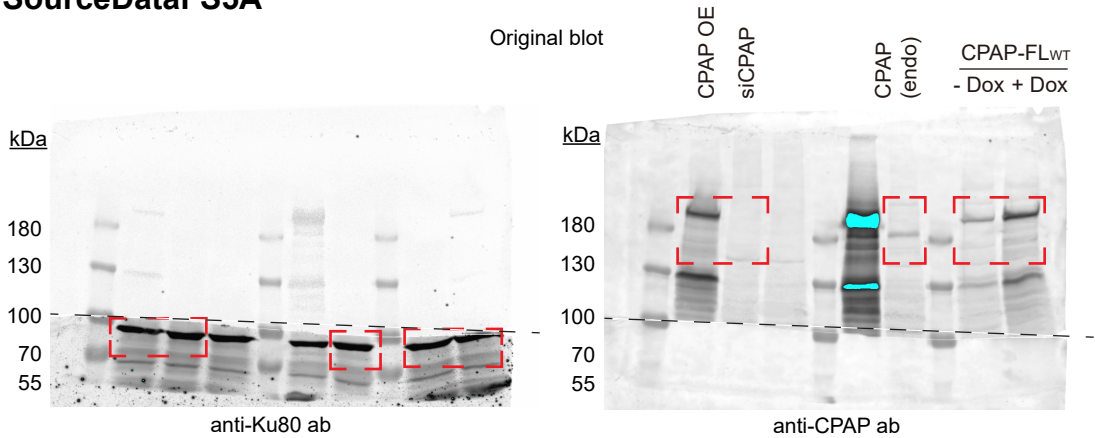

SourceDataFS5E

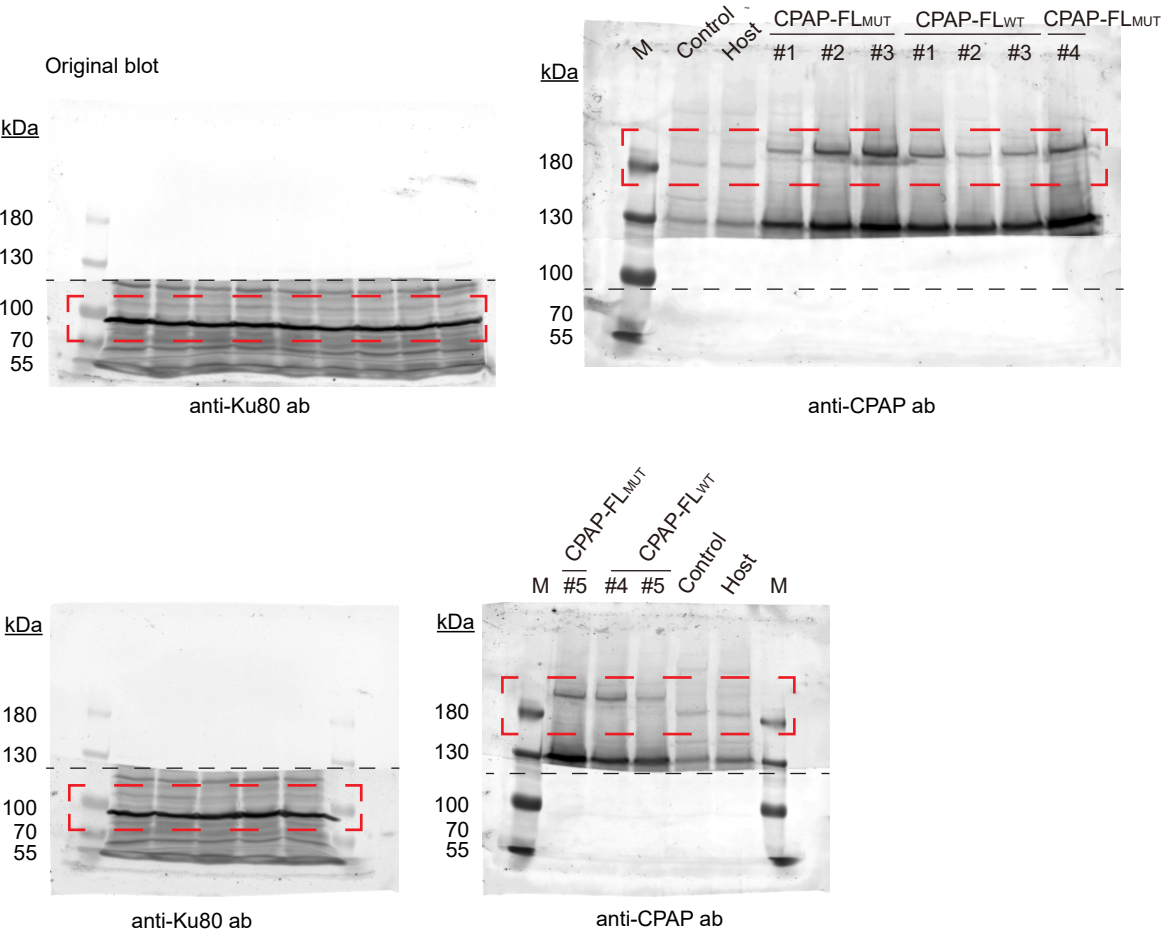

Supplement: SourceData FS5 — is the source file for Fig. S5. [file jcb_202406061_sourcedatafs5.pdf]
