# Supplementary material for: Centriolar cap proteins CP110 and CPAP control slow elongation of microtubule plus ends
Source: J Cell Biol. 2025 Jan 23;224(3):e202406061. doi: 10.1083/jcb.202406061 (PMC11756378; doi:10.1083/jcb.202406061)

Iyer et. al., - Source Data for gels and blots

SourceDataFS4

SourceDataFS4B

SDS-PAGE gel; (i) 1x CPAP-CC1 + 1x CP110-CC2

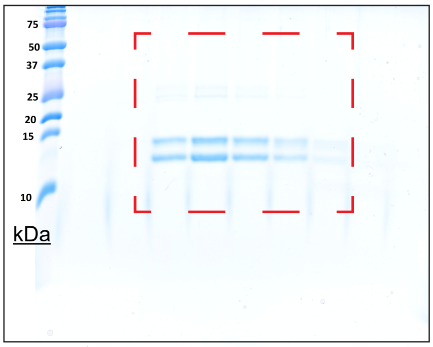

SDS-PAGE gel; (ii) 2x CPAP-CC1 + 1x CP110-CC2

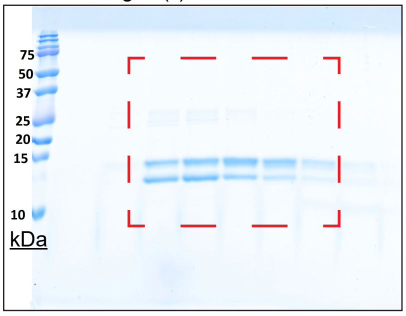

SDS-PAGE gel; (iii) 3x CPAP-Cc1 + 1x CP110-CC2

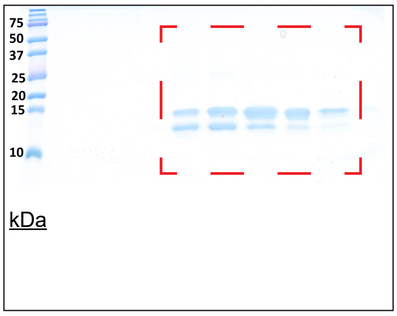

Supplement: SourceData FS4 — is the source file for Fig. S4. [file jcb_202406061_sourcedatafs4.pdf]
